# Predicting ventilator-associated lower respiratory tract infection outcomes using sequencing-based early microbiological response: a proof-of-concept prospective study

**DOI:** 10.3389/fcimb.2025.1547998

**Published:** 2025-05-12

**Authors:** Ji Zhou, Qian Qian, Chuwei Jing, Jing Liu, Danni Wang, Mingyue Wang, Yuchen Ding, Dejian Gu, Wenyin Xia, Lili Tao, Wenkui Sun

**Affiliations:** ^1^ Department of Respiratory and Critical Care Medicine, The First Affiliated Hospital with Nanjing Medical University, Nanjing, China; ^2^ Clinical Medicine School, Jiangsu Health Vocational College, Nanjing, China; ^3^ Clinical Medicine Research Institution, The First Affiliated Hospital with Nanjing Medical University, Nanjing, China; ^4^ Department of Medicine, Geneplus-Beijing Co., Ltd., Beijing, China; ^5^ Department of Laboratory Medicine, The First Affiliated Hospital with Nanjing Medical University, Nanjing, China; ^6^ Department of Pathology, Microbiology, and Immunology, Vanderbilt University Medical Center, Nashville, TN, United States

**Keywords:** NGS, VA-LRTI, VAP, *A. baumannii*, microbiological response

## Abstract

**Objectives:**

Ventilator-associated lower respiratory tract infections (VA-LRTIs) cause significant mortality. This study was assigned to explore the association between early microbiological responses defined by quantitative targeted amplicon-based next-generation sequencing (QtNGS) and clinical outcomes in patients with *Acinetobacter baumannii*-dominant VA-LRTI.

**Measurements and main results:**

A prospective observational study including 34 participants was conducted to assess the probability of predicting clinical outcomes using sequencing-based early microbiological response. Bronchoalveolar lavage fluids (BALFs) were collected at admission and 3 days post-treatment from these patients for QtNGS to determine the relative quantification ratio (RQR) of *A. baumannii*. Patients were categorized into survival (n=26) and non-survival (n=8) groups. The RQR was calculated as the quantification of *A. baumannii* determined by QtNGS after treatment to pretreatment. RQR significantly increased on day 4 in the non-survival group (median 5.285), and decreased in the survival group (median 0.1864). Receiver’s operation characteristic curves revealed that an RQR ≥1.41 was predictive of poor outcomes, with an area under the curve of 0.9471 (0.8759-1). The accuracy of the RQR determined by QtNGS was further evaluated by retesting the same specimen using digital droplet PCR, and the linear correlation was confirmed in the RQR calculated by two methods. The 23 patients with RQR<1.41 all survived for 28 days, whereas the survival rate for the 11 patients with RQR ≥1.41 was 27.27%. RQR was significantly and positively correlated with the length of ICU stay in survivors.

**Conclusions:**

The RQR of *A. baumannii* detected by QtNGS correlates with the prognosis of VA-LRTI patients.

## 1 Introduction

Lower respiratory tract infections (LRTIs) have emerged as a major global health concern, and rank the fourth leading cause of death worldwide, with about 2.4 million fatalities in 2019, as reported by the World Health Organization (Safiri et al., 2022). Ventilator-associated LRTIs (VA-LRTIs) pose a significant risk, and double the mortality rate compared to intubated patients without LRTI (Klompas, 2013; Vincent et al., 2009). Early evaluation of VA-LRTI treatment is crucial for timely modification of therapeutic strategies. However, accurately assessing the initial treatment response in these patients is still a challenge (Roberts et al., 2017). Current clinical assessments of VA-LRTI rely on a combination of clinical, radiological, and microbiological responses. Such evaluation is subjective, time-consuming and non-specific (Kalil et al., 2016). Studies have also investigated the use of biomarkers, such as procalcitonin (PCT) and C-reactive protein (CRP), for assessing treatment efficacy, but bring inconsistent findings (Póvoa et al., 2017; Huang et al., 2017). Moreover, accurately evaluating the microbiological response after treatment can help clinicians formulate appropriate antibiotic treatment courses (Bedi et al., 2021). Recently, more studies have focused on early microbiological response to assess early treatment response (Ceccato et al., 2022). However, how to evaluate pathogen load accurately remains a question.

The application of metagenomic next-generation sequencing (mNGS) has significantly improved the diagnosis of VA-LRTI (Cabibbe et al., 2018). To enhance the diagnostic capability of sequencing technology, targeted amplicon-based NGS (tNGS) involving specific probes designed to bind to targeted pathogen regions has been developed (Flurin et al., 2022). tNGS is effective in detecting multiple common respiratory pathogens at a quarter of the cost of mNGS (Gaston et al., 2022; Li et al., 2022). Various methods have been integrated with either mNGS or tNGS to quantify microbial load (Wanghu et al., 2024; Barlow et al., 2020). Zhou et al. introduced a sequencing-based quantitative method for estimating pathogen concentrations in samples (Zhou et al., 2022). Hauser et al. described the principles and methods of introducing internal controls in pathogen quantification by NGS (Hauser et al., 2023). These efforts have made it possible to assess microbiological response through sequence-based microbial quantification. However, the clinical significance of sequencing-based microbiological response has not yet been evaluated.

Our team utilized an innovative approach called quantitative tNGS (QtNGS) in our study to provide both qualitative and quantitative capabilities for pathogen detection. A prospective proof-of-concept study was conducted to explore the potential of QtNGS for assessing the bacterial load of *Acinetobacter baumannii* during treatment. Additionally, the associations between sequencing-based early microbiological responses and clinical outcomes were investigated.

## 2 Materials and methods

### 2.1 Study design and participants

This prospective study was carried out in the respiratory intensive care unit (RICU) of the First Affiliated Hospital of Nanjing Medical University, a leading regional medical center in Eastern China. Totally 542 patients admitted to the RICU between April 1, 2023 and Sep 30, 2024 were screened for participation. The inclusion criteria were: (1) aged 18 years or older; (2) meeting the diagnostic criteria for VA-LRTI; (3) *A. baumannii* identified within 72 hours prior to enrollment or confirmed through bronchoalveolar lavage fluid (BALF) microbiological testing post-preliminary screening. The exclusion criteria were: (1) refusal of patients or families to participate in the study; (2) inability to obtain BALF samples at enrollment or 3 days after enrollment; (3) receiving antimicrobial treatment for more than 3 days and demonstrating substantial improvement at the time of screening. The outcomes of the enrolled patients were evaluated at 28 days post-enrollment.

**Figure 1:**
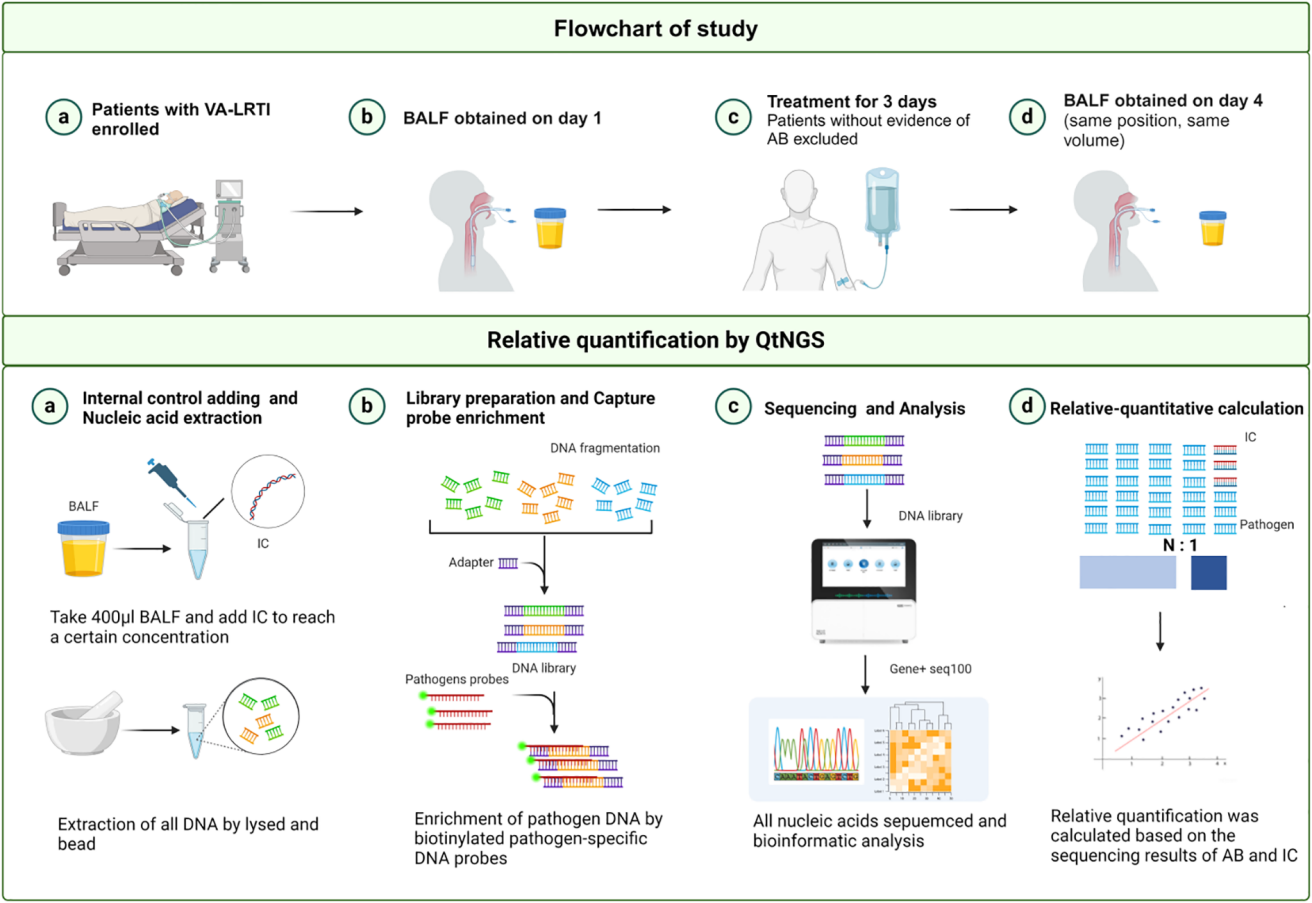
Flowchart and overview of the QtNGS procedure. VA-LRTI, ventilator-associated lower respiratory tract infection; BALF, bronchoalveolar lavage fluid; IC, internal control. Created with BioRender.com.

After the screening, bronchoscopy was performed promptly to obtain BALFs for bacterial culture and QtNGS. In cases where no etiology was defined during the initial screening, bronchoscopy was also performed for BALF collection. The BALF samples were then processed for bacterial culture or other diagnostic tests as determined by the attending physician’s discretion. Extra BALF samples were frozen at -80°C. If subsequent results confirmed *A. baumannii* infection, the patient was included, and the frozen BALF samples were analyzed using QtNGS. The identification of *A. baumannii* as the causative pathogen was independently verified by two investigators, Zhou and Sun. QtNGS showed *A. baumannii* was predominant in 32 of the 34 included patients, but was the second most abundant in the remaining 2 patients. A follow-up bronchoscopy was conducted 3 days after enrollment by targeting the same lung segment and by the same operator to ensure consistency. The treatment of the enrolled patients did not intervene across the study period. The attending physician decided on medication based on each patient’s condition. The flowchart of the study is outlined in **Figure 1**.

Definition:

*A. baumannii*-dominant VA-LRTI was defined as (Harrigan et al., 2021): (1) suspicion for respiratory infection was indicated via bacterial culture of respiratory specimens; (2) *A. baumannii* was isolated in the clinical microbiology laboratory; (3) vital signs or laboratory results indicated an infection (fever or white blood cell (WBC) count outside normal range). Immunocompromised status was defined as any of the following signs (Peng et al., 2021): (1) long-term treatment with steroids (>0.3 mg/g/d of prednisone equivalent for >3 weeks) or other immunosuppressant drugs; (2) chemotherapy during the last month; (3) hematological malignancy; (4) solid-organ transplantation during the last months; (5) inherited or acquired severe immunodeficiency virus infection.

### 2.2 The QtNGS experimental process

#### 2.2.1 Sample extraction and library construction

Given the risk of alignment errors with microbial genes that may lead to false positives or false negatives, we selected a segment of the *Arabidopsis thaliana* gene as an internal control. This internal control gene fragment was synthetically prepared and formulated into a solution at a concentration of 1.55 x 10^10 GEq/ml ± 10%. During the detection phase, 2.6 μL of the internal control solution was mixed with 400 μL of BALF, forming a final sample with an internal control concentration about 10^8 GEq/mL. The samples were extracted according to a reported procedure (Li et al., 2023). A library was prepared using a HieffNGS^®^ C37P4 OnePot cDNA & gDNA Library Prep Kit (from Yeasen, Shanghai, China) following the manufacturer’s instructions. Then the library was incubated with GenePlus-designed probes for 4 hours to completely capture target pathogens, which were then prepared into DNA nanoballs (DNBs). Pathogens that could be captured by the probes are listed in **Supplementary Table 1**.

#### 2.2.2 Sequencing and data processing

Sequencing was carried out on a GenePlus Seq-100 sequencing platform (GenePlus-Beijing) with 100-bp single-end read sequencing, aiming for a target depth of 5 million reads as the workflow. The sequenced data were then analyzed using the self-built database of GenePlus, and the number of reads of *A. baumannii* per million data volume was annotated (target-RPMCR). The number of internal control reads was annotated as IC-RPMCR. Finally, the relative quantification of *A. baumannii* was calculated by comparing target-RPMCR with IC-RPMCR as follows:


$$\text{RQ}=\frac{\text{target-RPMCR}}{\text{IC-RPMCR}}*10^{\wedge }8$$

The digital droplet PCR (ddPCR) procedure was as follows:

One milliliter of a sample was centrifuged at 12000 rpm for 2 min. Then the pellet was resuspended in 200 μL of lysozyme, and added with glass beads. The suspension was digested and fragmented for 10 min. After that, DNA was extracted using a nucleic acid extraction kit (Invensys, Hangzhou, China). Pathogens and antimicrobial resistance genes were detected using seven assay panels with a ddPCR system (Pilot Gene Technology Company, Hangzhou, China). The target pathogens detected here are shown in **Supplementary Table 2**.

### 2.3 Statistical analysis

Categorical and ordinal variables were described using frequencies and percentages. Normally distributed data were represented in mean (standard deviation); otherwise, median and interquartile ranges were used. To identify significant differences between groups, various statistical tests were utilized based on the nature and distribution of the data. These tests included the Mann–Whitney nonparametric test for continuous variables not following a normal distribution, Pearson’s chi-square test for categorical variables, paired t-test for paired comparisons, and two-tailed Fisher’s exact test for contingency tables. A p value less than 0.05 was considered as statistical significance. Receiver’s operating characteristic (ROC) curves were plotted to assess the prognostic value of the variables. The area under the curve (AUC) was calculated, and the optimal cutoff values were determined to estimate the sensitivity and specificity of the variables. All the statistical analyses were performed using SPSS 24 (SPSS Inc., Chicago, Illinois, USA).

## 3 Results

### 3.1 Patient samples

**Figure 2:**
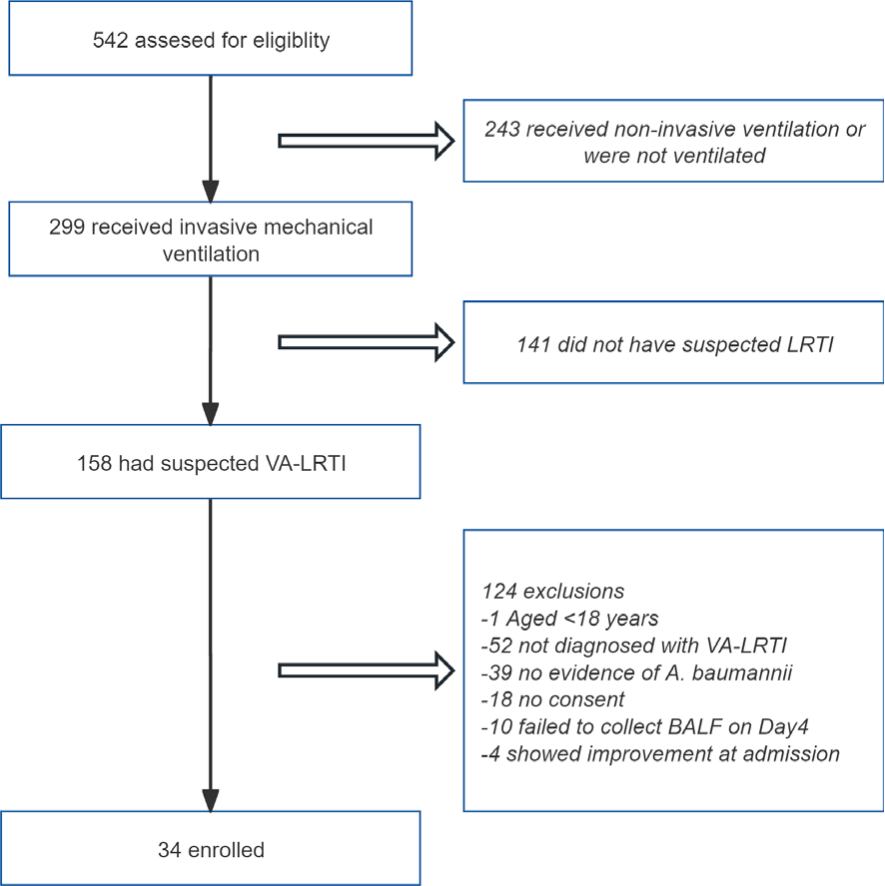
Flowchart of the enrollment process for the study. LRTI, lower respiratory tract infection; BALF, bronchoalveolar lavage fluid.

A total of 542 patients admitted to the RICU between April 1, 2023 and Sep 30, 2024 were screened for inclusion. Of them, 243 patients without receiving mechanical ventilation, and 141 patients not fulfilling the diagnostic criteria for VA-LRTI were excluded. After the use of other inclusion and exclusion criteria, 34 patients with VA-LRTI predominantly caused by *A. baumannii* were ultimately included. The patient screening process is depicted in **Figure 2**.

### 3.2 *In vitro* relative quantification of *A. baumannii* by QtNGS

**Figure 3:**
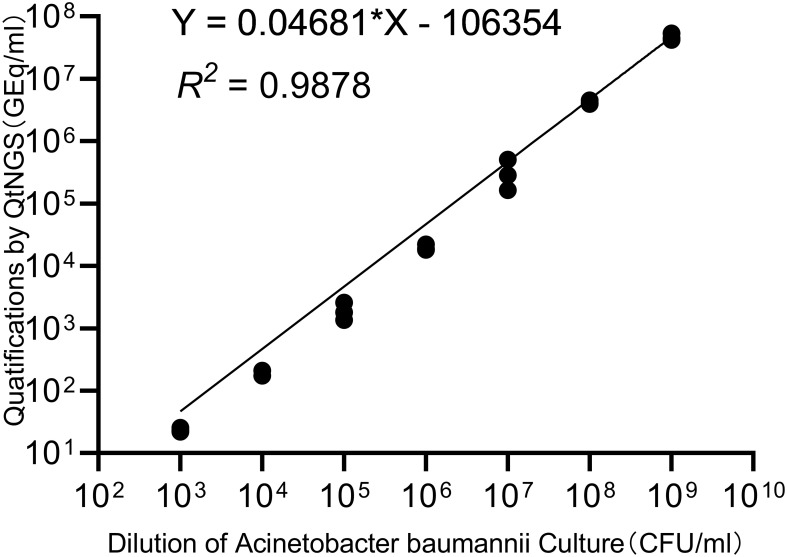
Correlation of quantification by QtNGS and bacterial concentration. The quantification of bacteria by QtNGS was linearly correlated with the A. baumannii concentration.

*A. baumannii* strains were separated and cultured from clinical samples, and the concentration of the bacterial suspension was estimated by measuring the absorbance at 260 nm. The bacterial suspension was then serially diluted for quantification by QtNGS in triplicate. The correlation between the quantification by QtNGS and the bacterial concentration was evaluated. The relative quantification measured by QtNGS was linearly correlated with the bacterial concentration (**Figure 3**), with a calculated slope of 0.04681 (95% CI: 0.04432 to 0.04931). The determination coefficient (R^2^) was 0.9878.

### 3.3 Patient characteristics

A total of 34 patients were enrolled over a 28-day follow-up period. Their demographic and clinical characteristics are detailed in **Table 1**. Among these patients, 8 died during the follow-up period, resulting in a mortality rate of 23.53%. The average age of the cohort was 71 years, and the majority were male (23 patients, 67.65%). Comorbidities were common, as 13 patients (38.24%) had a comorbidity score of 2 or higher. The clinical characteristics of the patients were compared between the survival and non-survival groups. Significantly lower PCT levels were observed in the survival group than in the non-survival group at admission (*p=0.009*). Lower WBC count and higher PaO2/FiO2 ratio were found in the survival group after treatment. Other indicators, including the SOFA score, mCPIS score, and IL-6 and CRP levels, did not significantly differ either before treatment or after treatment.

**Table 1:** Clinical characteristics of included patients.

Characteristic	All (n=34)	Survival Group (n=26)	Non-Survival Group (n=8)	P valve
Age, years	71 (60.75-76.25)	69.5 (60.75-75.25)	72.5 (58.25-83)	0.647^a^
Male sex	23 (67.65%)	17 (65.38%)	6 (75%)	1.000^b^
McCabe and Jackson Score for commodities ≥2	13 (38.24%)	9 (34.62%)	4 (50%)	0.679^b^
lmmuno depression	3 (8.82%)	2 (7.69%)	1 (12.5%)	1.000^b^
Length of stay in ICU, days	14 (10-20.25)	14 (10.75-21.25)	16.5 (7.5-17.75)	0.735^a^
Length of stay in hospital, days	14 (10-22.5)	14 (10.75-24.75)	16.5 (7.5-19.25)	0.647^a^
Variables at admission				
APACHE II	13.62 (5.22)	13.77 (5.43)	13.13 (4.79)	0.765^c^
SOFA	6.44 (2.9)	6.12 (2.85)	7.5 (3.02)	0.244^c^
White blood cell count, Giga/L	10.7 (5.57-14.31)	8.63 (5.33-12.93)	12.8 (11.14-14.5)	0.130^a^
PaO2/FiO2 ratio, mmHg	231.11 (171.75-336.88)	235.56 (174.5-367.64)	227.78 (165.25-296.74)	0.765^a^
mCPIS	5.07 (1.91)	4.91 (1.93)	5.5 (1.93)	0.463^c^
CRP,mg/L	86.75 (47.93-157)	77.05 (44.6-157)	104.05 (53.2-279.52)	0.436^a^
PCT,ng/mL	0.61 (0.25-2.46)	0.33 (0.17-1.26)	1.58 (0.75-10.73)	***0.009*** ^a^
IL-6, pg/mL	47.52 (22.02-132.3)	40.42 (21.35-104.4)	105.23 (44.94-150.68)	0.177^a^
Variables at Day4				
APACHE II	12.53 (4.69)	12.23 (4.8)	13.5 (4.44)	0.511^c^
SOFA	6.07 (2.97)	6.14 (3.09)	5.83 (2.79)	0.827^c^
White blood cell count, Giga/L	8.36 (8.74-11.16)	7.46 (5.31-10.45)	11.69 (9.28-14.28)	***0.019*** ^a^
PaO2/FiO2 ratio, mmHg	272.67 (128.18)	295.83 (130.1)	186.65 (78.4)	***0.043*** ^c^
mCPIS	5.67(1.96)	5.35 (1.93)	6.57 (1.9)	0.160^c^
CRP,mg/L	75.61 (71.55)	66.76 (44.63)	104.68 (127.11)	0.226^c^
PCT,ng/mL	0.45 (0.16-0.94)	0.38 (0.14-0.92)	0.82 (0.45-3.56)	0.102^a^
IL-6, pg/mL	40.16 (18.93-74)	38.81 (16.01-64.88)	62.61 (38.77-152.13)	0.118^a^

P Values were calculated by Manne Whitney U test (a), Fisher’s exact test (b) or student’s t-test (c).

### 3.4 Early microbiological response and clinical outcomes

**Figure 4:**
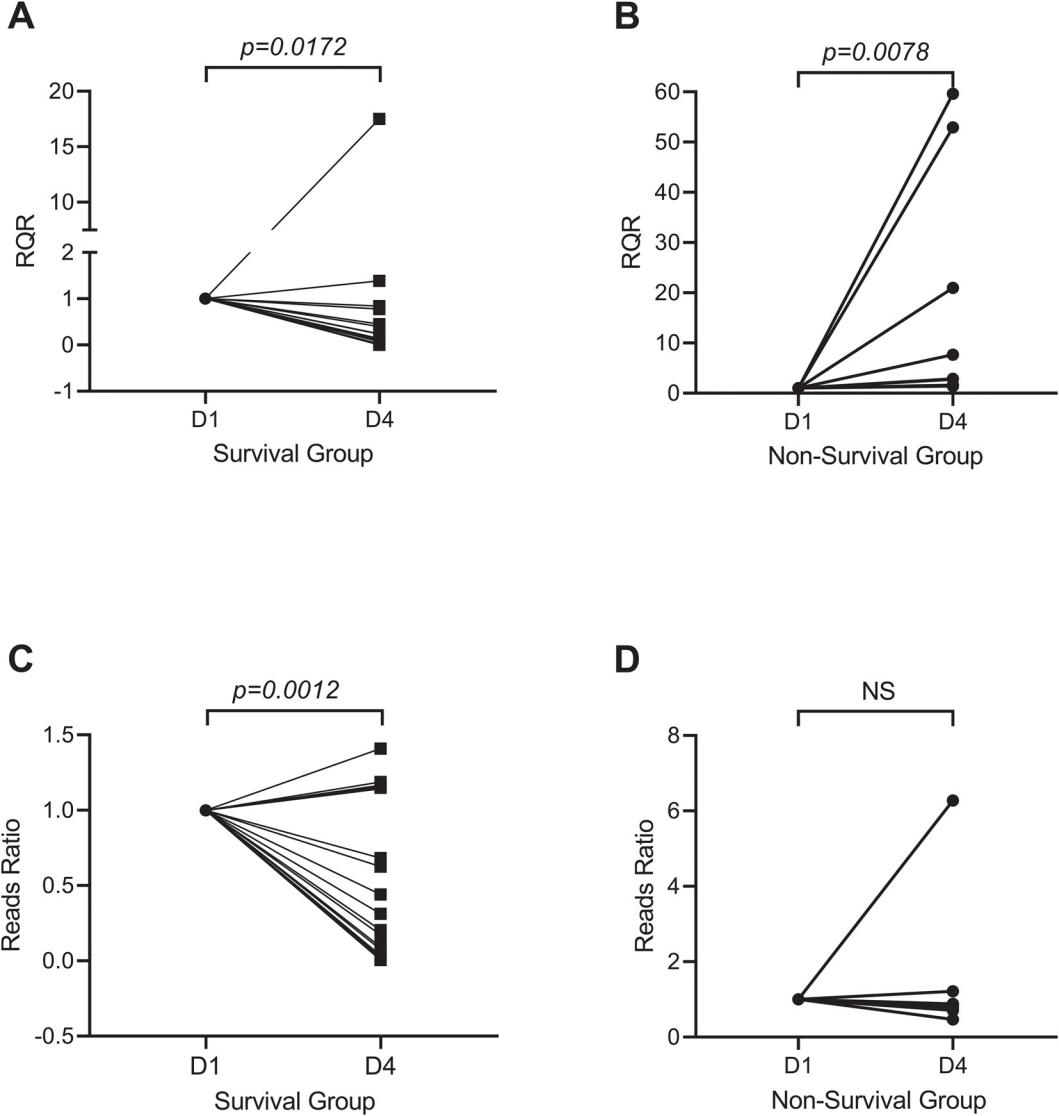
Microbiological response of *A. baumannii* after treatment. Survivors **(A, C)** showed reduced RQR (Relative Quantitative Ratio; median 0.14, p=0.0172) and RR (Reads Ratio; median 0.24, p=0.0012), whereas non-survivors **(B, D)** exhibited elevated RQR (median 5.29, p=0.0078) and stable RR.

We evaluated the microbiological response of each patient by calculating the reads ratio (RR) and the relative quantification ratio (RQR). The RR was calculated as the ratio of *A. baumannii* reads obtained posttreatment to those obtained pretreatment. Similarly, the RQR was calculated as the ratio of *A. baumannii* quantification posttreatment to pretreatment. RR and RQR were standardized to 100% for all patients on the first day of enrollment. In the survival group, both RQR and RR significantly decreased following treatment (**Figures 4A, C**). The median RR after treatment was 0.2398 in the survival group, while the median RQR was 0.1380. In the non-survival group, the RR did not significantly change after treatment. However, the RQR significantly increased after treatment (**Figure 4B**, *p=0.0078*). The median RQR for the non-surviving group was 5.285 after treatment. RQR was also significantly and positively correlated with the length of ICU stay in all survivors (**Supplementary Figure S1**, *p* = 0.0099). The difference of RQR posttreatment between the survival group and non-survival group is not affected by antibiotic resistance, as no significant differences in resistance were observed between the *A. baumannii* strains isolated from the two groups (**Supplementary Table 3**).

**Figure 5:**
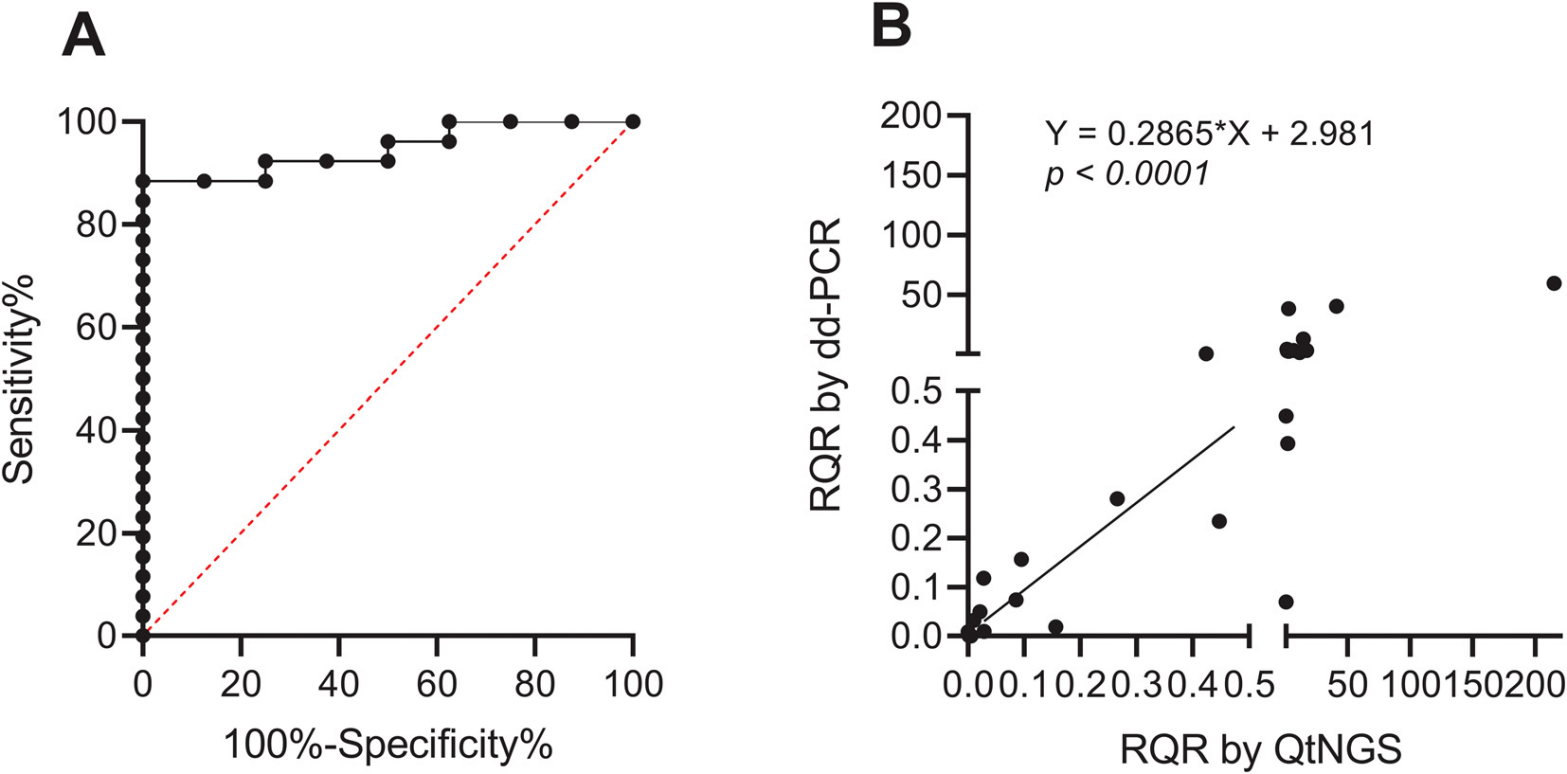
ROC curve and verification of RQR. **(A)** ROC curve of the RQR for predicting clinical outcomes (AUC = 0.9471). **(B)** Linearly correlation between RQRs determined by dd-PCR and QtNGS.

ROC curve analysis was used to determine the optimal cutoff value of the RQR in predicting clinical outcomes (**Figure 5A**). The analysis revealed a cutoff value of 1.41 for the RQR in effectively distinguishing between different clinical outcomes. AUC for this ROC curve was 0.9471 (0.8759 to 1). No death was observed in patients with RQR< 1.41 during the follow-up period. In contrast, 8 out of the 11 patients (72.7%) with elevated RQR (RQR ≥ 1.41) succumbed within 28 days, showing a substantially larger mortality rate.

To verify the accuracy of the RQR measured through QtNGS, we retested BALF specimens collected during the study period from the first included 28 patients using dd-PCR (except for patient #1, due to insufficient specimen volume). A significant linear correlation of RQRs detected by QtNGS and ddPCR was noted (*p* < 0.0001) for *A. baumannii* (**Figure 5B**).

## 4 Discussion

To the best of our knowledge, this study represents the first investigation of RQR in patients with VA-LTRI through quantitative sequencing during the early phase of antibiotic treatment. The RQR identified in this study provides new insight for early evaluation of treatment response in VA-LRTI patients.

Inaccurate evaluation of treatment efficacy can lead to prolonged use of inappropriate empiric antibiotic treatment and to increased mortality (Kadri et al., 2021). The current ERS/ESICM/ESCMID/ALAT guidelines do not provide indications for routine follow-up of microbiological studies. Instead, clinical assessment, including tracheobronchial secretion volume and assessment of the purulence of tracheobronchial secretions, is recommended for VA-LRTI patients receiving antibiotic treatment, aiming to predict clinical response at 72–96 hours (Torres et al., 2017). Previous research has shed light on the predictive value of follow-up cultures for ventilation-associated pneumonia. A’Court et al. reported that patients with a positive clinical response to antibiotic treatment showed significantly decreased mean colony counts in nondirected bronchial lavage (NBL) after antibiotic initiation (A’Court et al., 1993). In contrast, NBL in patients showing progressive clinical deterioration did not significantly decrease (A’Court et al., 1993). Prats et al. assessed the effects of antibiotic treatment on the results of protected specimen brushing in patients with ventilator-associated pneumonia (Prats et al., 2002). The amount of bacteria significantly decreased within 12 hours after effective antibiotic treatment (Prats et al., 2002). As reported, the identification of new microorganisms after treatment in patients was associated with increased mortality and a prolonged duration of mechanical ventilation (Ceccato et al., 2022). However, the application of bacterial culture in assessing early microbiological response is limited by its long turnaround time. Additionally, empirical antimicrobial treatments can reduce the sensitivity of bacterial cultures (Gadsby et al., 2016). Moreover, quantification by culture was not precise. Significant correlation was found in the concentrations determined from NGS and quantitative PCR, but not in the concentrations detected by quantitative NGS and microbial culture (Hauser et al., 2023).

Recent studies have attempted to evaluate pathogen loads using sequencing. A sequencing-based tuberculosis molecular bacterial load assay was demonstrated effective in describing differences in pathogen clearance between patients with recurrent tuberculosis and those who responded effectively to treatment (Ntinginya et al., 2022). However, the reads in the existing mNGS reports cannot be directly used as the concentration of a pathogen. The commonly-used protocol of NGS reveals only the relative microbial distribution of the sample, but not the information regarding absolute quantification (Kallastu et al., 2023). The US Food and Drug Administration recommends the use of an internal control, which is typically a “foreign sequence” coextracted and co-analyzed during sample sequencing (Hauser et al., 2023). The quantification of pathogen load is facilitated by introducing an internal control. tNGS is more sensitive in detecting pathogens than mNGS. Moreover, tNGS offers cost advantages and is less time-consuming (Yi et al., 2024). QtNGS was ultimately chosen for this study. Our study revealed a strong association between follow-up RQR identified by QtNGS and mortality in patients with *A. baumannii-*dominated VA-LRTI, as the mortality rate in patients with RQR ≥ 1.41 was up to 72.73%. The RQR based on QtNGS is an individualized and precise indicator of pathogen clearance in the early stages of treatment. This indicator results jointly from the interactions among the patient, the pathogen, and the medical intervention. It facilitates individualized antibiotic course management and antibiotic adjustment.

DdPCR as the most effective technology for determining the absolute DNA load does not need controls and provides precise concentrations (Kallastu et al., 2023). However, ddPCR was not used to quantify bacterial load in our study, because this technology requires the design of specific primers and presupposes an accurate prediction of potential pathogens, which are similar to all other PCR-based methods (Yi et al., 2024). Although only *A. baumannii* was evaluated in this study, the sequencing-based technology chosen here guarantees broad application for quantifying other predicted or unpredicted pathogens in the future. Nonetheless, we used ddPCR to further verify the accuracy of the RQR determined by QtNGS, and found a linear correlation between the RQRs determined by QtNGS and ddPCR.

A well-recognized limitation of QtNGS for quantification of pathogens is that the measurement is based on the amount of genomic DNA present in the specimen. Therefore, QtNGS cannot determine if the pathogen is viable or not, and the presence of pathogen reads does not suggest continuing infection. However, we hypothesize that the reads ratio of a certain pathogen decreases with effective treatment, and use RQR to describe the relative changes in pathogen quantification after initial treatment. We indeed found RQR decreased in the survival group, but increased in the non-survival group.

Despite promising results, our study has limitations. This study was conducted in a single center and included only 34 patients. Thus, the findings may not be generalizable to other clinical settings. Future studies shall be aimed to validate these findings in a larger, multicenter cohort. We evaluated only patients with *A. baumannii* VA-LRTI. Whether RQR is applicable to other pathogens for treatment response evaluation requires further studies.

In conclusion, this study introduces a novel method for evaluation of early microbiological response and provides promising clinical observation results. QtNGS represents a promising approach for assessing treatment efficacy, estimating prognosis, and guiding adjustments in patients with VA-LRTI.

## Data Availability

The datasets presented in this study can be found in online repositories. The names of the repository/repositories and accession number(s) can be found in the article/**Supplementary Material**.

## References

[B1] A’Court C. H. Garrard C. S. Crook D. Bowler I. Conlon C. Peto T.. (1993). Microbiological lung surveillance in mechanically ventilated patients, using non-directed bronchial lavage and quantitative culture. Q. J. Med. 86, 635–648. doi: 10.1093/qjmed/86.10.635 8255961

[B2] Barlow J. T. Bogatyrev S. R. Ismagilov R. F. (2020). A quantitative sequencing framework for absolute abundance measurements of mucosal and lumenal microbial communities. Nat. Commun. 11, 2590. doi: 10.1038/s41467-020-16224-6 32444602 PMC7244552

[B3] Bedi P. Cartlidge M. K. Zhang Y. Turnbull K. Donaldson S. Clarke A.. (2021). Feasibility of shortening intravenous antibiotic therapy for bronchiectasis based on bacterial load: a proof-of-concept randomised controlled trial. Eur. Respir. J. 58, 2004388. doi: 10.1183/13993003.04388-2020 34112732

[B4] Cabibbe A. M. Walker T. M. Niemann S. Cirillo D. M. (2018). Whole genome sequencing of Mycobacterium tuberculosis. Eur. Respir. J. 52, 1801163. doi: 10.1183/13993003.01163-2018 30209198

[B5] Ceccato A. Dominedò C. Ferrer M. Martin-Loeches I. Barbeta E. Gabarrús A.. (2022). Prediction of ventilator-associated pneumonia outcomes according to the early microbiological response: a retrospective observational study. Eur. Respir. J. 59, 2100620. doi: 10.1183/13993003.00620-2021 34475230

[B6] Flurin L. Wolf M. J. Mutchler M. M. Daniels M. L. Wengenack N. L. Patel R. (2022). Targeted metagenomic sequencing-based approach applied to 2146 tissue and body fluid samples in routine clinical practice. Clin. Infect. Dis. Off. Publ. Infect. Dis. Soc Am. 75, 1800–1808. doi: 10.1093/cid/ciac247 PMC9662179 35362534

[B7] Gadsby N. J. Russell C. D. McHugh M. P. Mark H. Conway Morris A. Laurenson I. F.. (2016). Comprehensive molecular testing for respiratory pathogens in community-acquired pneumonia. Clin. Infect. Dis. Off. Publ. Infect. Dis. Soc Am. 62, 817–823. doi: 10.1093/cid/civ1214 PMC4787606 26747825

[B8] Gaston D. C. Miller H. B. Fissel J. A. Jacobs E. Gough E. Wu J.. (2022). Evaluation of metagenomic and targeted next-generation sequencing workflows for detection of respiratory pathogens from bronchoalveolar lavage fluid specimens. J. Clin. Microbiol. 60, e0052622. doi: 10.1128/jcm.00526-22 35695488 PMC9297812

[B9] Harrigan J. J. Abdallah H. O. Clarke E. L. Oganisian A. Roy J. A. Lautenbach E.. (2021). Respiratory microbiome disruption and risk for ventilator-associated lower respiratory tract infection. Clin. Infect. Dis. Off. Publ. Infect. Dis. Soc Am. 74, 1564–1571. doi: 10.1093/cid/ciab678 PMC9630883 34363467

[B10] Hauser S. Lazarevic V. Tournoud M. Ruppé E. Santiago Allexant E. Guigon G.. (2023). A metagenomics method for the quantitative detection of bacterial pathogens causing hospital-associated and ventilator-associated pneumonia. Microbiol. Spectr. 11, e0129423. doi: 10.1128/spectrum.01294-23 37889000 PMC10715005

[B11] Huang H.-B. Peng J.-M. Weng L. Wang C.-Y. Jiang W. Du B. (2017). Procalcitonin-guided antibiotic therapy in intensive care unit patients: a systematic review and meta-analysis. Ann. Intensive Care 7, 114. doi: 10.1186/s13613-017-0338-6 29168046 PMC5700008

[B12] Kadri S. S. Lai Y. L. Warner S. Strich J. R. Babiker A. Ricotta E. E.. (2021). Inappropriate empirical antibiotic therapy for bloodstream infections based on discordant *in-vitro* susceptibilities: a retrospective cohort analysis of prevalence, predictors, and mortality risk in US hospitals. Lancet Infect. Dis. 21, 241–251. doi: 10.1016/S1473-3099(20)30477-1 32916100 PMC7855478

[B13] Kalil A. C. Metersky M. L. Klompas M. Muscedere J. Sweeney D. A. Palmer L. B.. (2016). Management of adults with hospital-acquired and ventilator-associated pneumonia: 2016 clinical practice guidelines by the infectious diseases society of America and the American thoracic society. Clin. Infect. Dis. Off. Publ. Infect. Dis. Soc Am. 63, e61–e111. doi: 10.1093/cid/ciw353 PMC4981759 27418577

[B14] Kallastu A. Malv E. Aro V. Meikas A. Vendelin M. Kattel A.. (2023). Absolute quantification of viable bacteria abundances in food by next-generation sequencing: Quantitative NGS of viable microbes. Curr. Res. Food Sci. 6, 100443. doi: 10.1016/j.crfs.2023.100443 36691592 PMC9860258

[B15] Klompas M. (2013). Complications of mechanical ventilation–the CDC’s new surveillance paradigm. N. Engl. J. Med. 368, 1472–1475. doi: 10.1056/NEJMp1300633 23594002

[B16] Li X. Liu Y. Li M. Bian J. Song D. Liu C. (2023). Epidemiological investigation of lower respiratory tract infections during influenza A (H1N1) pdm09 virus pandemic based on targeted next-generation sequencing. Front. Cell. Infect. Microbiol. 13. doi: 10.3389/fcimb.2023.1303456 PMC10755937 38162581

[B17] Li S. Tong J. Liu Y. Shen W. Hu P. (2022). Targeted next generation sequencing is comparable with metagenomic next generation sequencing in adults with pneumonia for pathogenic microorganism detection. J. Infect. 85, e127–e129. doi: 10.1016/j.jinf.2022.08.022 36031154

[B18] Ntinginya N. E. Bakuli A. Mapamba D. Sabiiti W. Kibiki G. Minja L. T.. (2022). Tuberculosis molecular bacterial load assay reveals early delayed bacterial killing in patients with relapse. Clin. Infect. Dis. Off. Publ. Infect. Dis. Soc Am. 76, e990–e994. doi: 10.1093/cid/ciac445 PMC9907486 35717643

[B19] Peng J.-M. Du B. Qin H.-Y. Wang Q. Shi Y. (2021). Metagenomic next-generation sequencing for the diagnosis of suspected pneumonia in immunocompromised patients. J. Infect. 82, 22–27. doi: 10.1016/j.jinf.2021.01.029 33609588

[B20] Póvoa P. Martin-Loeches I. Ramirez P. Bos L. D. Esperatti M. Silvestre J.. (2017). Biomarkers kinetics in the assessment of ventilator-associated pneumonia response to antibiotics - results from the BioVAP study. J. Crit. Care 41, 91–97. doi: 10.1016/j.jcrc.2017.05.007 28502892

[B21] Prats E. Dorca J. Pujol M. Garcia L. Barreiro B. Verdaguer R.. (2002). Effects of antibiotics on protected specimen brush sampling in ventilator-associated pneumonia. Eur. Respir. J. 19, 944–951. doi: 10.1183/09031936.02.00239302 12030737

[B22] Roberts K. L. Micek S. T. Juang P. Kollef M. H. (2017). Controversies and advances in the management of ventilator associated pneumonia. Expert Rev. Respir. Med. 11, 875–884. doi: 10.1080/17476348.2017.1378574 28891372

[B23] Safiri S. Mahmoodpoor A. Kolahi A.-A. Nejadghaderi S. A. Sullman M. J. M. Mansournia M. A.. (2022). Global burden of lower respiratory infections during the last three decades. Front. Public Health 10. doi: 10.3389/fpubh.2022.1028525 PMC9869262 36699876

[B24] Torres A. Niederman M. S. Chastre J. Ewig S. Fernandez-Vandellos P. Hanberger H.. (2017). International ERS/ESICM/ESCMID/ALAT guidelines for the management of hospital-acquired pneumonia and ventilator-associated pneumonia: Guidelines for the management of hospital-acquired pneumonia (HAP)/ventilator-associated pneumonia (VAP) of the European Respiratory Society (ERS), European Society of Intensive Care Medicine (ESICM), European Society of Clinical Microbiology and Infectious Diseases (ESCMID) and Asociación Latinoamericana del Tórax (ALAT). Eur. Respir. J. 50, 1700582. doi: 10.1183/13993003.00582-2017 28890434

[B25] Vincent J.-L. Rello J. Marshall J. Silva E. Anzueto A. Martin C. D.. (2009). International study of the prevalence and outcomes of infection in intensive care units. JAMA 302, 2323–2329. doi: 10.1001/jama.2009.1754 19952319

[B26] Wanghu H. Li Y. Huang J. Pu K. Guo F. Zhong P.. (2024). A novel synthetic nucleic acid mixture for quantification of microbes by mNGS. Microb. Genomics 10, 1199. doi: 10.1099/mgen.0.001199 PMC10926700 38358316

[B27] Yi X. Lu H. Liu X. He J. Li B. Wang Z.. (2024). Unravelling the enigma of the human microbiome: Evolution and selection of sequencing technologies. Microb. Biotechnol. 17, e14364. doi: 10.1111/1751-7915.14364 37929823 PMC10832515

[B28] Zhou H. Ouyang C. Han X. Shen L. Ye J. Fang Z.. (2022). Metagenomic sequencing with spiked-in internal control to monitor cellularity and diagnosis of pneumonia. J. Infect. 84, e13–e17. doi: 10.1016/j.jinf.2021.09.018 34600934

