# Supplementary figures and images for: Predicting ventilator-associated lower respiratory tract infection outcomes using sequencing-based early microbiological response: a proof-of-concept prospective study
Source: Front Cell Infect Microbiol. 2025 May 12;15:1547998. doi: 10.3389/fcimb.2025.1547998 (PMC12104225; doi:10.3389/fcimb.2025.1547998)

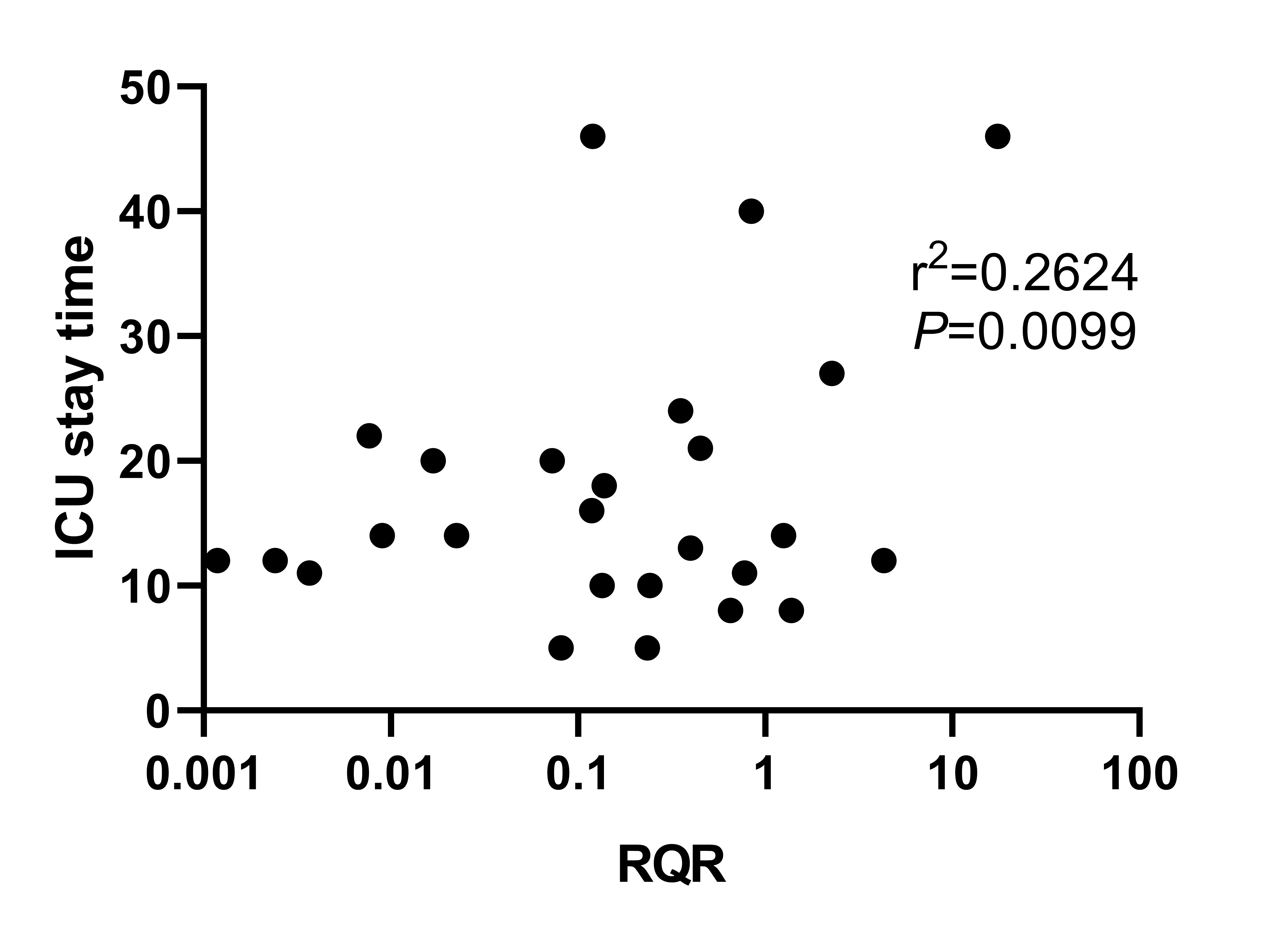

Supplement: Supplementary file 1 [file Image1.tif]
