# Supplementary material for: Predicting ventilator-associated lower respiratory tract infection outcomes using sequencing-based early microbiological response: a proof-of-concept prospective study
Source: Front Cell Infect Microbiol. 2025 May 12;15:1547998. doi: 10.3389/fcimb.2025.1547998 (PMC12104225; doi:10.3389/fcimb.2025.1547998)
Supplement: Supplementary file 2 [file Table1.doc]

**Supplementary Table 1.** Pathogens detected by QtNGS.

| **No.** | **Pathogen** |
| --- | --- |
| 1 | Middle East respiratory syndrome coronavirus |
| 2 | Severe acute respiratory syndrome-related coronavirus |
| 3 | SARS-CoV-2 |
| 4 | Rhinovirus A |
| 5 | Rhinovirus B |
| 6 | Rhinovirus C |
| 7 | Influenza C virus |
| 8 | Enterovirus A71 |
| 9 | Enterovirus D68 (EV-D68) |
| 10 | Human alphaherpes virus 1 |
| 11 | Human alphaherpes virus 2 |
| 12 | Rubella virus |
| 13 | Human Respiratory syncytial virus A |
| 14 | Human Respiratory syncytial virus B |
| 15 | Influenza A virus(H1N1) |
| 16 | Influenza A virus(H3N2) |
| 17 | Influenza A virus(H5N1) |
| 18 | Influenza A virus(H7N9) |
| 19 | Influenza A virus(H9N2) |
| 20 | Human betaherpes virus 5 |
| 21 | Coxsackievirus A2 |
| 22 | Coxsackievirus A4 |
| 23 | Coxsackievirus A5 |
| 24 | Coxsackievirus A6 |
| 25 | Coxsackievirus A7 |
| 26 | Coxsackievirus A8 |
| 27 | Coxsackievirus A9 |
| 28 | Coxsackievirus A10 |
| 29 | Coxsackievirus A16 |
| 30 | Coxsackievirus B1 |
| 31 | Coxsackievirus B2 |
| 32 | Coxsackievirus B3 |
| 33 | Coxsackievirus B4 |
| 34 | coxsackievirus B5 |
| 35 | Measles virus |
| 36 | Human herpesvirus 4 |
| 37 | Human bocavirus 1 |
| 38 | Human respirovirus 1 |
| 39 | Human orthorubulavirus 2 |
| 40 | Human respirovirus 3 |
| 41 | Human orthorubulavirus 4 |
| 42 | Human coronavirus 229E |
| 43 | Human coronavirus HKU1 |
| 44 | Human coronavirus NL63 |
| 45 | Human coronavirus OC43 |
| 46 | Human betaherpesvirus 6 |
| 47 | Human metapneumovirus |
| 48 | Parechovirus A |
| 49 | Human parechovirus 1 |
| 50 | Human parechovirus 3 |
| 51 | Mumps virus |
| 52 | Human alphaherpesvirus 3 |
| 53 | Human parvovirus B19 |
| 54 | Influenza B virus |
| 55 | Echovirus E3 |
| 56 | Echovirus E6 |
| 57 | Echovirus E9 |
| 58 | Echovirus E16 |
| 59 | Echovirus E17 |
| 60 | Echovirus E25 |
| 61 | Echovirus E30 |
| 62 | Human adenovirus 1 |
| 63 | Human adenovirus 2 |
| 64 | Human adenovirus B3 |
| 65 | Human adenovirus E4 |
| 66 | Human adenovirus 5 |
| 67 | Human adenovirus 7 |
| 68 | Human adenovirus 14 |
| 69 | Human adenovirus 55 |
| 70 | Human adenovirus 11 |
| 71 | Human adenovirus 21 |
| 72 | Human adenovirus 31 |
| 73 | Human adenovirus 34 |
| 74 | Human adenovirus 35 |
| 75 | Orthohantavirus |
| 76 | Hantaan orthohantavirus |
| 77 | Seoul orthohantavirus |
| 78 | Elizabethkingia anophelis |
| 79 | Nocardia brasiliensis |
| 80 | Corynebacterium diphtheriae |
| 81 | Bordetella pertussis |
| 82 | Cronobacter sakazakii |
| 83 | Mycobacterium intracellulare |
| 84 | Acinetobacter baumannii |
| 85 | Nocardia beijingensis |
| 86 | Coxiella burnetii |
| 87 | Pseudomonas mallei |
| 88 | Klebsiella variicola |
| 89 | Corynebacterium propinquum |
| 90 | Mycobacterium xenopi |
| 91 | Streptococcus pyogenes |
| 92 | Klebsiella aerogenes |
| 93 | Klebsiella oxytoca |
| 94 | Yersinia pseudotuberculosis |
| 95 | Yersinia enterocolitica |
| 96 | Treponema denticola |
| 97 | Bacteroides thetaiotaomicron |
| 98 | Phocaeicola vulgatus |
| 99 | Bacteroides fragilis |
| 100 | Escherichia coli |
| 101 | Listeria monocytogenes |
| 102 | Gordonia bronchialis |
| 103 | Pasteurella dagmatis |
| 104 | Pasteurella pneumotropica |
| 105 | Pasteurella multocida |
| 106 | Aggregatibacter actinomycetemcomitans |
| 107 | Klebsiella pneumoniae |
| 108 | Streptococcus pneumoniae |
| 109 | Chlamydia pneumoniae |
| 110 | Mycoplasma pneumoniae |
| 111 | Tsukamurella pulmonis |
| 112 | Enterococcus faecalis |
| 113 | Shigella |
| 114 | Plesiomonas shigelloides |
| 115 | Citrobacter braakii |
| 116 | Citrobacter amalonaticus |
| 117 | Citrobacter freundii |
| 118 | Shewanella alga |
| 119 | Shewanella putrefaciens |
| 120 | Bordetella parapertussis |
| 121 | Haemophilus parainfluenzae |
| 122 | Mycobacterium leprae |
| 123 | Mycobacterium parascrofulaceum |
| 124 | Haemophilus parahaemolyticus |
| 125 | Mycobacterium simiae |
| 126 | Mycobacterium gordonae |
| 127 | Nocardia arthritidis |
| 128 | Mycobacteroides chelonae |
| 129 | Dialister pneumosintes |
| 130 | Bartonella henselae |
| 131 | Clostridium perfringens |
| 132 | Clostridium tetani |
| 133 | Streptococcus porcinus |
| 134 | Streptococcus mitis |
| 135 | Tropheryma whipplei |
| 136 | helicobacter pylori |
| 137 | Bordetella holmesii |
| 138 | Corynebacterium pseudodiphtheriticum |
| 139 | Corynebacterium pseudotuberculosis |
| 140 | Corynebacterium jeikeium |
| 141 | Mycobacterium tuberculosis complex |
| 142 | Metamycoplasma hominis |
| 143 | Ureaplasma urealyticum |
| 144 | Staphylococcus aureus |
| 145 | Kingella kingae |
| 146 | Moraxella catarrhalis |
| 147 | Mycobacterium kansasii |
| 148 | Citrobacter koseri |
| 149 | Corynebacterium ulcerans |
| 150 | Bacillus cereus |
| 151 | Nocardia veterana |
| 152 | Burkholderia pseudomallei |
| 153 | Klebsiella quasipneumoniae |
| 154 | Rickettsia rickettsii |
| 155 | Brucella abortus |
| 156 | Haemophilus influenzae |
| 157 | Mycobacterium scrofulaceum |
| 158 | Acinetobacter calcoaceticus |
| 159 | Acinetobacter lwoffii |
| 160 | Brucella melitensis |
| 161 | Legionella maceachernii |
| 162 | Mycobacterium malmoense |
| 163 | Tatlockia micdadei |
| 164 | Morganella morganii |
| 165 | Achromobacter xylosoxidans |
| 166 | Nocardia cyriacigeorgica |
| 167 | Nocardia transvalensis |
| 168 | Neisseria meningitidis |
| 169 | Mycobacterium avium complex (MAC) |
| 170 | Mycobacterium colombiense |
| 171 | Mycobacterium avium |
| 172 | Mycobacterium abscessus |
| 173 | Nocardia abscessus |
| 174 | Mycolicibacterium fortuitum |
| 175 | Proteus penneri |
| 176 | Nocardia farcinica |
| 177 | Chryseobacterium indologenes |
| 178 | Acinetobacter pittii |
| 179 | Proteus vulgaris |
| 180 | Proteus mirabilis |
| 181 | Eikenella corrodens |
| 182 | Brucella canis |
| 183 | Alcaligenes faecalis |
| 184 | Ochrobactrum anthropi |
| 185 | Cardiobacterium hominis |
| 186 | Haemophilus haemolyticus |
| 187 | Arcanobacterium haemolyticum |
| 188 | Salmonella enterica subsp. enterica serovar Paratyphi A |
| 189 | Salmonella enterica subsp. enterica serovar Paratyphi B |
| 190 | Salmonella enterica subsp. enterica serovar Paratyphi C |
| 191 | Salmonella enterica subsp. enterica serovar Typhimurium |
| 192 | Salmonella enterica subsp. enterica serovar Enteritidis |
| 193 | Salmonella enterica |
| 194 | Chlamydia trachomatis |
| 195 | Sphingomonas paucimobilis |
| 196 | Borrelia burgdorferi |
| 197 | Borrelia duttoni |
| 198 | Borrelia recurrentis |
| 199 | Leptospira interrogans |
| 200 | Leptospira |
| 201 | Pseudomonas stutzeri |
| 202 | Enterococcus faecium |
| 203 | Legionella bozemanii |
| 204 | Legionella dumoffii |
| 205 | Legionella pneumophila |
| 206 | Stenotrophomonas maltophilia |
| 207 | Aggregatibacter aphrophilus |
| 208 | Aeromonas hydrophila |
| 209 | Yersinia pestis |
| 210 | Providencia rettgeri |
| 211 | Providencia alcalifaciens |
| 212 | Providencia stuartii |
| 213 | Bacillus anthracis |
| 214 | Pseudomonas gladioli |
| 215 | Streptococcus dysgalactiae subsp. equisimilis |
| 216 | Pseudomonas aeruginosa |
| 217 | Francisella tularensis |
| 218 | Nocardia otitidiscaviarum |
| 219 | Aeromonas caviae |
| 220 | Ureaplasma parvum |
| 221 | Aeromonas veronii bv. sobria |
| 222 | Corynebacterium striatum |
| 223 | Legionella waltersii |
| 224 | Streptococcus agalactiae |
| 225 | Bartonella vinsonii |
| 226 | Bartonella bacilliformis |
| 227 | Bartonella quintana |
| 228 | Bordetella hinzii |
| 229 | Nocardia nova |
| 230 | Streptococcus constellatus |
| 231 | Streptococcus anginosus |
| 232 | Burkholderia cepacia complex |
| 233 | Burkholderia cepacia |
| 234 | Burkholderia cenocepacia |
| 235 | Burkholderia multivorans |
| 236 | Rickettsia tsutsugamushi |
| 237 | Actinomyces israelii |
| 238 | Acinetobacter nosocomialis |
| 239 | Acinetobacter haemolyticus |
| 240 | Acinetobacter junii |
| 241 | Enterobacter cloacae |
| 242 | Enterobacter cloacae complex |
| 243 | Chlamydia abortus |
| 244 | Chlamydia psittaci |
| 245 | Pseudomonas putida |
| 246 | Pseudomonas fluorescens |
| 247 | Serratia marcescens |
| 248 | Legionella longbeachae |
| 249 | Bordetella bronchiseptica |
| 250 | Raoultella planticola |
| 251 | Streptococcus intermedius |
| 252 | Brucella suis |
| 253 | Chromobacterium violaceum |
| 254 | Mycobacterium marinum |
| 255 | Mycobacterium ulcerans |
| 256 | Mycobacterium haemophilum |
| 257 | Nocardia asteroides |
| 258 | Nocardia puris |
| 259 | Nocardia wallacei |
| 260 | Nocardia asiatica |
| 261 | Rhodococcus equi |
| 262 | Elizabethkingia meningoseptica |
| 263 | Rickettsia typhi |
| 264 | Rickettsia prowazekii |
| 265 | Lichtheimia corymbifera |
| 266 | Rhizomucor pusillus |
| 267 | Rhizomucor miehei |
| 268 | Trichosporon asahii |
| 269 | Paracoccidioides brasiliensis |
| 270 | Candida albicans |
| 271 | Aspergillus versicolor |
| 272 | Coccidioides posadasii |
| 273 | Coccidioides immitis |
| 274 | Lomentospora prolificans |
| 275 | Candida parapsilosis |
| 276 | [Candida] glabrata |
| 277 | Candida tropicalis |
| 278 | Candida auris |
| 279 | Fusarium solani |
| 280 | Cryptococcus laurentii |
| 281 | Naganishia albida |
| 282 | Cryptococcus gattii |
| 283 | Aspergillus oryzae |
| 284 | Aspergillus nidulans |
| 285 | Aspergillus flavus |
| 286 | Cunninghamella bertholletiae |
| 287 | Histoplasma capsulatum |
| 288 | Fusarium oxysporum |
| 289 | Scedosporium apiospermum |
| 290 | Mucor circinelloides |
| 291 | Schizophyllum commune |
| 292 | Talaromyces marneffei |
| 293 | Rhizopus oryzae |
| 294 | Exophiala dermatitidis |
| 295 | Blastomyces dermatitidis |
| 296 | Sporothrix schenckii |
| 297 | Aspergillus terreus |
| 298 | Rhizopus microsporus |
| 299 | Cryptococcusneoformans |
| 300 | Curvularia lunata |
| 301 | Apophysomyces elegans |
| 302 | Aspergillusfumigatus |
| 303 | Pneumocystisjirovecii |
| 304 | Mucor indicus |
| 305 | Syncephalastrum racemosum |
| 306 | Mucor racemosus |

**Supplementary Table 2.** Pathogens and AMR genes detection panels for ddPCR assay.

| **Assay panel** | **Target pathogens** |
| --- | --- |
| **PilotBac-1** | A. baumannii, E. coli, K. pneumonia, P. aeruginosa, |
| **PilotBac-2** | E. faecalis, E. faecium, S. aureus, S. pneumoniae |
| **PilotBac-3** | S. capitis, S. haemolyticus, S. hominis, S. epidermidis |
| **PilotBac-4** | E. cloacae, P. mirabilis, S. marcescens, S. maltophilia |
| **PilotFungi-1** | C. albicans, C. glabrata, C. parapsilosis, C. tropicalis |
| **PilotAMR-1** | bla_KPC_, mecA, vanA, vanB |
