# Supplementary material for: Predicting ventilator-associated lower respiratory tract infection outcomes using sequencing-based early microbiological response: a proof-of-concept prospective study
Source: Front Cell Infect Microbiol. 2025 May 12;15:1547998. doi: 10.3389/fcimb.2025.1547998 (PMC12104225; doi:10.3389/fcimb.2025.1547998)
Supplement: Supplementary file 3 [file Table2.docx]

**Supplementary Table 2.** Pathogens and AMR genes detection panels for ddPCR assay.

| **Assay panel** | **Target pathogens** |
| --- | --- |
| **PilotBac-1** | A. baumannii, E. coli, K. pneumonia, P. aeruginosa, |
| **PilotBac-2** | E. faecalis, E. faecium, S. aureus, S. pneumoniae |
| **PilotBac-3** | S. capitis, S. haemolyticus, S. hominis, S. epidermidis |
| **PilotBac-4** | E. cloacae, P. mirabilis, S. marcescens, S. maltophilia |
| **PilotFungi-1** | C. albicans, C. glabrata, C. parapsilosis, C. tropicalis |
| **PilotAMR-1** | bla_KPC_, mecA, vanA, vanB |
