# Supplementary material for: Predicting ventilator-associated lower respiratory tract infection outcomes using sequencing-based early microbiological response: a proof-of-concept prospective study
Source: Front Cell Infect Microbiol. 2025 May 12;15:1547998. doi: 10.3389/fcimb.2025.1547998 (PMC12104225; doi:10.3389/fcimb.2025.1547998)
Supplement: Supplementary file 4 [file Table3.docx]

Supplementary Table 3：Antibiotic Resistance Characteristics of *Acinetobacter baumannii*.

| Antibiotic | Non-survivors (n=8) | Survivors (n=26) | P value |
| --- | --- | --- | --- |
| Meropenem | 7/8 (87.5%) | 18/26 (69.2%) | 0.413 |
| Imipenem | 7/8 (87.5%) | 18/26 (69.2%) | 0.413 |
| Piperacillin-tazobactam | 6/8 (75.0%) | 12/26 (46.2%) | 0.232 |
| Cefoperazone-sulbactam | 6/8 (75.0%) | 14/26 (53.8%) | 0.422 |
| Amikacin | 7/8 (87.5%) | 17/26 (65.4%) | 0.386 |
| Tigecycline | 6/8 (75.0%) | 13/26 (50.0%) | 0.257 |
| Colistin | 2/8 (25.0%) | 6/26 (23.1%) | 1 |
| Levofloxacin | 8/8 (100%) | 24/26 (92.3%) | 1 |
| MDR* | 7/8 (87.5%) | 18/26 (69.2%) | 0.413 |

*Multidrug-resistance (MDR) was defined as acquired non-susceptibility to at least one agent in three or more antimicrobial categories.
